# An Integrated Analytical Approach for the Characterization of Probiotic Strains in Food Supplements

**DOI:** 10.3390/nu14235085

**Published:** 2022-11-29

**Authors:** Veronica Bolzon, Massimo Pesando, Michela Bulfoni, Alessandro Nencioni, Emanuele Nencioni

**Affiliations:** 1 Biofarma Group Srl., Via Castelliere 2, 33036 Udine, Italy; 2 Department of Medicine, University of Udine, 33100 Udine, Italy; 3 IBSA Institut Biochimique SA, Via del Piano 29, CH-6915 Pambio Noranco, Switzerland

**Keywords:** probiotics, real-time quantitative PCR (RT-qPCR), food supplements, flow-cytometry, plate count enumeration, quality control (QC), food industry, ICH guidelines, method validation

## Abstract

Research surrounding health benefits from probiotics is becoming popular because of the increasing demand for safer products with protective and therapeutic effects. Proven benefits are species- or genus-specific; however, no certified assays are available for their characterization and quantification at the strain level in the food supplement industry. The objective of this study was to develop a strain-specific Real-time quantitative polymerase chain reaction (RT-qPCR)-based method to be implemented in routine tests for the identification and quantification of *Bifidobacterium longum*, *Bifidobacterium animalis* spp. *lactis*, *Lactobacillus paracasei*, *Lactobacillus rhamnosus*, *Lactobacillus casei*, *Bifidobacterium breve*, *Lactobacillus acidophilus*, *Lactobacillus plantarum*, and *Lactobacillus helveticus*, starting from a powder mixture of food supplements. The method optimization was carried out in combination with flow cytometry to compare results between the two strategies and implement the analytical workflow with the information also regarding cell viability. These assays were validated in accordance with the International Council for Harmonization of Technical Requirements for Pharmaceuticals for Human Use (ICH) criteria using the plate count enumeration as the gold standard reference. Briefly, probiotic DNAs were extracted from two powder food supplements. Strain-specific primers targeting unique sequence regions of 16S RNA were identified and amplified by RT-qPCR. Primers were tested for specificity, sensitivity, and efficiency. Both RT-qPCR and flow-cytometry methods described in our work for the quantification and identification of *Lactobacillus* and *Bifidobacterium* strains were specific, sensitive, and precise, showing better performances with respect to the morphological colony identification. This work demonstrated that RT-qPCR can be implemented in the quality control workflow of commercial probiotic products giving more standardized and effective results regarding species discrimination.

## 1. Introduction

*Lactobacilli* spp. and *Bifidobacteria* spp. are widely used as probiotics in human food supplements [1,2]. The identification of specific probiotic strains is important to discriminate their different biological properties [1,3,4]. In fact, the potential health benefits of probiotics, such as the production of anti-microbial metabolites, synthesis of B-group vitamins, anti-obesity, anti-diabetic effect, cholesterol-lowering effect, oxidative stress, and down-regulation, seem to vary from strain to strain [1,5,6,7].

Despite the large impact of the probiotic industry and the wide use of probiotics as food supplements, the most common assays currently employed for their identification and characterization are based on classical microbiological methods [3,8,9,10]. In general, these methods are time consuming, operator dependent, and often, the phenotypic identification of strains based on morphology may be due to their misidentifications [4,11,12]. Morphology screening for colony differentiation is difficult to standardize and is often applied to incorrect discrimination of strains [4,13].

Recently, for the species-specific identification of strains, genomic-based profiling by real-time quantitative polymerase chain reaction (RT-qPCR) analysis has been proposed as a key metric in the probiotic industrial field to ensure their correct definition [14,15,16]. The RT-qPCR method combines the qualitative identification of strains with the absolute quantification of bacteria obtainable by creating a standard calibration curve of known quantities of starting templates [16,17,18]. The highlight of RT-qPCR allows the detection of several bacterial species simultaneously in food, environmental matrices, cosmetics, feces, and other complex matrices [4,5,16,17]. Considering its high sensitivity, RT-qPCR enables the quantification of microorganisms with low abundance within a sample without the necessity of a preliminary enrichment step in culture [16,18,19,20]. The key point in RT-qPCR development is primer design that needs to specifically target the strain of interest [3,15,21]. Furthermore, both the efficiency and the accuracy of the RT-qPCR reaction depend on DNA quality and quantity [22,23]. The main problem for reaching a good quality DNA is the removal of PCR-inhibitory elements derived from the food supplement matrix. Different commercial kits are available to obtain an efficient DNA extraction. In our previous work, we have already tested and validated the best method for DNA isolation and purification from probiotics contained in complex matrices [24].

In pursuit of a rapid, convenient, and cost-effective method for monitoring bacterial viability, costs and time are important aspects to take into consideration. The ideal procedure should present a cost-effective and rapid management to accurately determine the absolute count of viable cells. Culture procedures are considered expensive and time consuming with low statistical accuracy. Costs are associated mainly with selective culture media and broths for bacterial growth. Approximately 4 to 10 plates are needed to obtain at least a duplicate cell count of an unknown sample. Furthermore, large differences among plate counts, derived from inter-operator variability, result in large standard deviations (about 20–30% variability). Using plating techniques, the time to results is often 2–5 days of incubation time, making product release long [25,26].

With flow cytometry and molecular biology approaches, the testing time is relatively short—within 2 h for results with the first technology and within 8 h with the second one—with a significant gain in “worker effort” (e.g., man-hour). In fact, man-hour is the basis for measuring the cost of professional people and their contribution to productivity. Furthermore, flow cytometry shows less variability with a mean of 10% standard deviation. Reagents are relatively cheap, considering the number of tests performable per hour (up to 48 in 1 h) [27,28,29,30].

RT-qPCR is considered expensive because of reagent composition and instruments. The high number of tests enables substantial savings in reagent costs, technical burden, and time to generate laboratory results [31,32,33].

The implementation of new technologies is cost-effective and an effective long-term solution in the management of the high number of samples per day and charged to the operator [30,34,35]. Compared to classical microbiological methods, flow cytometry and RT-qPCR provide the optimal balance of cost in quality control screening scenarios.

The species of both *Lactobacillus* and *Bifidobacterium* generis are taxonomically very complex for their phenotypical diversity. Nevertheless, 16S rRNA gene sequences are too similar to be easily distinguished. In particular, closely related species within the *L. acidophilus* group (*L. acidophilus*, *L. gallinarum*, and *L. helveticus*), the *L. casei* group *(L. casei*, *L. paracasei*, and *L. rhamnosus*), the *L. plantarum* group (*L. plantarum*, *L. paraplantarum*, and *L. pentosus*), and the *L. sakei* group (*L. sakei*, *L. curvatus*, and *L. graminis*) are notoriously difficult to distinguish due to the high morphological homology [22,36,37].

To make the assay more robust, the combination with another detection method, such as flow cytometry, has gained prominence in the industry [19,38]. The main goal of flow cytometry is the total enumeration of viable, dead, and damaged bacterial cells in a relatively short time [23,28,38,39]. The assessment of cell viability inherits the advantages of fluorescent dyes. In particular, bacterial viability is differentiated by membrane permeability properties and is based on a dual staining procedure, such as thiazole orange (TO) or SYTO 9 (Thermofisher Scientific, Waltham, MA, USA) and propidium iodide (PI). Both dyes intercalate with nucleic acids. TO/SYTO 9 can cross all bacterial cell membranes, while PI can only enter cells with disrupted membranes allowing differentiation between live and dead cells [28,38].

Together, genomics and flow cytometry have revolutionized strain-specific microbial detection, allowing us to specifically quantify viable probiotics and give them a strain genomic identity [14,15,36]. In recent years, the publication by the International Standard in according with ISO 19344 (IDF 232) has suggested a new method for the selective enumeration of active lactic acid bacteria/or total units by flow cytometry to assess the quality of fermented products (ISO 19344 2015) [13,24,40,41]. High-throughput analysis using a flow-cytometric based-count gave the advantages of a lower variation, a reduction in testing time, the differentiation between active and total cells, and quantification of the fraction of active cells per total cells [23,28,30].

In this study, we describe the development of a combined analytical assay based on flow cytometry and strain-specific RT-qPCRs for the identification and absolute quantification of probiotics in food supplements.

The detection of *Bifidobacterium longum*, *Bifidobacterium animalis* spp. *lactis*, *Lactobacillus paracasei*, *Lactobacillus rhamnosus*, *Lactobacillus casei*, *Bifidobacterium breve*, *Lactobacillus acidophilus*, *Lactobacillus plantarum,* and *Lactobacillus helveticus* strains have been carried out from two different powder commercial food supplements.

In this work, flow cytometry in combination with RT-qPCR was tested for the first time as a specific quantification and qualification quality control method for *Lactobacillus* and *Bifidobacterium*-based products. Primers were tested for specificity with a set of non-target probiotic strains while sensitivity, the limit of detection (LOD), and efficiency were optimized using scalar dilutions. The presence and quantity of probiotics were also determined by plate count enumeration as the reference gold standard method recognized for quality assurance of production. To improve the quality of our study, a multiplex qPCR approach based on TaqMan probes (Thermofisher Scientific, Waltham, MA, USA) was designed. In fact, the simultaneous amplification of more than one target sequence in a single tube allows an even faster screening of different probiotic strains. We performed single reactions per bacterium species to validate the method step by step. In the future, the application of TaqMan probes (Thermofisher Scientific, Waltham, MA, USA) with better specificity will produce faster results in the quality control workflow.

## 2. Materials and Methods

### 2.1. Food Supplement Composition

Food supplements employed in this work are 2 anonymized commercial formulations of powder probiotics with different compositions. The probiotic powder composition of food supplement 1 was formulated with 7 species: commercial strain of *Bifidobacterium longum*, *Bifidobacterium animalis* spp. *lactis*, *Lactobacillus paracasei*, *Lactobacillus acidophilus*, *Lactobacillus plantarum*, *Bifidobacterium breve,* and *Lactobacillus helveticus*; while the probiotic powder composition of food supplement 2 was composed by 4 strains: *Bifidobacterium animalis* spp. *lactis* (≥85.2 × 10^9^ CFU/g), *Bifidobacterium breve* (≥10.8 × 10^9^ CFU/g), *Lactobacillus rhamnosus* (≥12 × 10^9^ CFU/g), and *Lactobacillus paracasei* (≥12 × 10^9^ CFU/g). Food supplement 1 was packaged in 2 different formats, premix and sachets, composed of the same probiotic mix but in different total quantities, ≥285 × 10^9^ CFU/g and 102 × 10^9^ CFU/g, respectively, without any discrimination between strain-specific counts.

Considering that no quantifications for each strain were available, food supplement 1 was employed to set the sensitivity and the specificity of the RT-qPCR reactions and to define the qualitative composition of the probiotic mix; instead, food supplement 2, characterized by declared strain enumeration, was used to optimize quantitative RT-qPCR reactions comparing results.

### 2.2. Growth Conditions and Plate Count Enumeration

Briefly, 1 g of each lyophilized probiotic was diluted in Maximum Recovery Diluent (MRD-Biolife Italiana, Rome, Italy), according to the Italian National Institute of Health (ISTISAN 08/36) guidelines [40]. Considering food supplement 1, a premix aliquot and 3 batches of sachets (A, B, and C) were tested, while for food supplement 2, 3 sachets of the same batch were analyzed. Culture of all strains was performed following the ISTISAN 08/36 for *Lactobacillus* spp. and ISO 29981:2012 for the *Bifidobacteria* spp., which allows discriminating probiotics on the bases of colony’s morphology [39,40].

**Figure 1:**
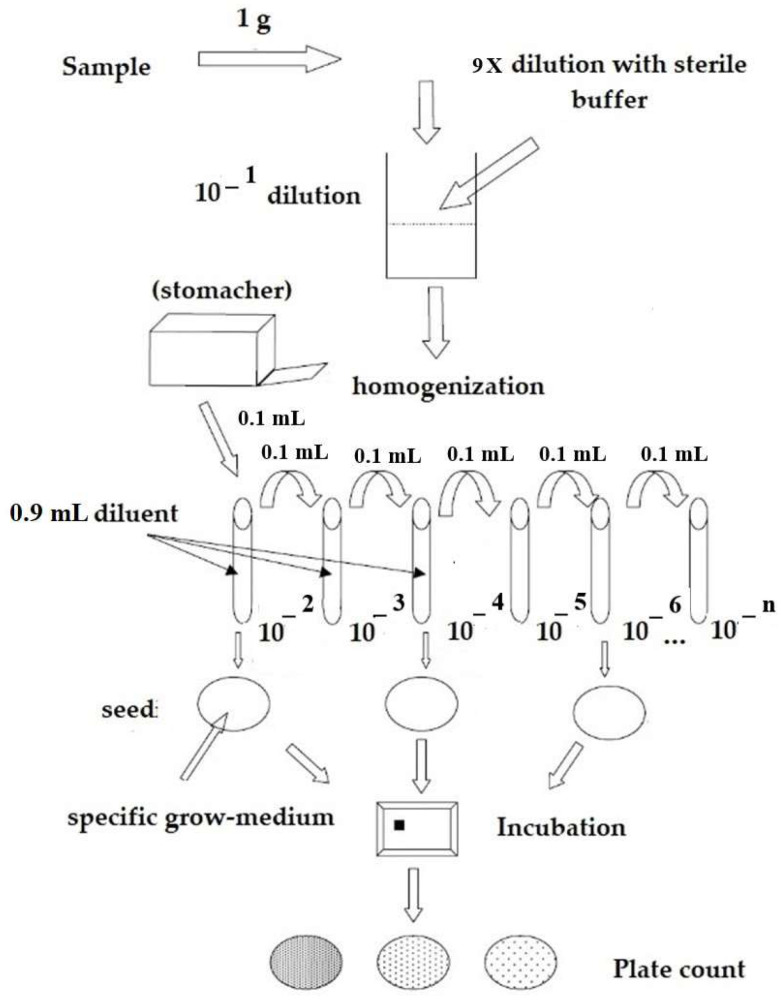
Representative flow chart of the operative workflow employed for plate count enumeration.

For plate seeding, samples were serially diluted, and the 10 × 10^−9^/10^−10^ dilutions of food supplement 1 were inoculated on Petri plates containing de Man, Rogosa, Sharpe agar (MRS—Biolife Italiana, Rome, Italy), added with 0.05% L-cystein hydrochloric acid (HCl) (Sigma Aldrich, Milwaukee, WI, USA) while for the food supplement 2 the 10 × 10^−8^/10^−9^/10^−10^ dilutions were inoculated on Petri plates containing de Man, Rogosa, Sharpe agar (MRS—Biolife Italiana, Rome, Italy), added with 0.05% L-cystein HCl (Sigma Aldrich, Milwaukee, WI, USA) and TOS-Propionate Agar base (TOS—Merck Millipore, Burlington, MA, USA), with MUP Selective Supplement, as appropriate, all in duplicates (Figure 1). Plates were incubated at 37 ± 2 °C for 48–72 h in anaerobiosis. Grown colonies have been counted in duplicate on two consecutive dilutions (two plates for 10 × 10^−9^ dilution and two plates for 10 × 10^−10^ dilution), where the expected number of colonies was between 3 and 300. The proportionality was verified with the calculation of the Kp (coverage factor for measurement uncertainty, as described by ISO 13005 [39,40]) and the G2 (chi square test).

Plate counts were performed, ensuring proportionality between the different dilutions tested. Results were expressed as colony-forming units per gram (CFU/g), in scientific notation, with 1 or 2 decimal numbers, considering the weighted average value.

### 2.3. Flow-Cytometry

Two-color staining using the BD Cell Viability Kit (BD Biosciences, San Jose, CA, USA) was performed on all probiotic suspensions of food supplements 1 and 2 to distinguish between live and dead cells by flow cytometry. First, the appropriate dilutions of samples were incubated for 30 min in the dark with 42 µmol/L of TO and then an additional 30 min with 4.3 mmol/L of PI. Properly conjugated isotype-matched dyes and unstained bacterial samples were used as negative controls (the same bacterial cells incubated without any dye). Stained samples were analyzed employing a FACS Verse (BD Biosciences, San Jose, CA, USA) flow cytometer. A total of 10,000 events were acquired per sample using an automatic compensation tool [41]. Data were analyzed with the FACSuite™ (BD Biosciences, San Jose, CA, USA) software, in the “Acquisition-to-Analysis” mode. A forward scatter (FSC) vs. side scatter (SSC) plot with a physical gate was designed to identify the bacterial population of interest. Then, another specific region in the fluorescence parameter 1 (FL1 (TO)) vs. fluorescence parameter 3 (FL3 (PI)) plot was gated to display the live/dead stain results. To determine the absolute count, expressed as UFC/g, the following equation was used:

(1) N=n×1000×d

(*n* = number of events per µL; *d* = dilution factor corresponding to the dilution).

### 2.4. DNA Extraction from Powder Probiotics 

Bacterial DNA was extracted starting from 1 mL of the 10^−1^ dilution of 6 samples of the premix and from 6 sachets belonging to 3 batches (two per each batch A, B, and C) for food supplement 1 and from 3 sachets belonging to one batch for the food supplement 2. DNA was isolated using the Power Soil Kit (Qiagen GmbH, Hilden, Germany), according to the manufacturer’s instructions using a Qiacube Connect extractor (Qiagen GmbH, Hilden, Germany). Prior to starting with the automated kit protocol, samples were incubated with Lysozyme (50 mg/mL) for 45 min at 37 °C and then homogenized for 10 s at 1000 g using Precellys^®^ SK38 bead beating tubes (Peqlab Biotechnology GmbH, Erlangen, Germany) and the Precellys^®^ 24-Dual homogenizer (Peqlab Biotechnology GmbH, Erlangen, Germany). The volume of extraction and the elution solution was chosen according to the manufacturer’s instruction. Isolated DNA was visualized by agarose gel electrophoresis, and concentration was determined by NanoDrop ND-1000 spectrophotometer (Peqlab Biotechnology GmbH, Erlangen, Germany).

### 2.5. Strain-Specific Primer Design and qPCR Assay

Strain-specific PCR primers were designed or modified from literature and checked for sequence similarity by the primer BLAST tool of the National Center of Biotechnology Information (NCBI) [32,33,34,35,36]. A set of primers for each species was tested for sensitivity, specificity, and efficiency using an annealing temperature gradient-qPCR. The following conditions were applied: initial denaturation at 95 °C for 3 min followed by 40 cycles at 95 °C for 30 s, annealing at 58 °C–63 °C for 15 s, elongation at 72 °C for 30 s, and a final step at 72 °C for 5 min. To ensure that the correct PCR product was amplified, a melting curve analysis was added at the end of the PCR program using the following gradient thermal protocol: 65 °C–95 °C with a delta temperature of 0.5 °C every 5 s. Amplification was carried out in a 15 µL final volume containing 1 × qPCR ssoAdvance SYBR Master Mix (Biorad, Hercules, CA, USA), 500 nM of each primer, and 2 µL target DNA using a QIA-quant 96 instrument. The best-performing primers employed on food supplement 1 for the sole qualitative discrimination are listed in Table 1.

Primer sequences used for the absolute quantification of species in food supplement 2 are reported in Table 2.

For each amplification reaction, the melting temperature (Tm) and cycle-threshold (Ct) values were computed. For the absolute quantification of DNA copies, a standard curve composed of points, representative of different concentrations, was built. The sensitivity of RT-qPCR was determined by the lowest concentration tested (10 × 10^−11^), while the specificity and the threshold signal were defined using a no-probiotic DNA or other probiotic DNAs, and a no template control as references.

### 2.6. Optimization of RT-qPCR

For the experimental set-up, optimization of assay parameters, such as primer concentration, annealing temperature, and amplification efficiency conditions, was performed. The specificity of primers was studied with other 3 distinct bacteria strains (no-probiotic DNA) and with the DNA of the other probiotic strains compounding food supplement mixture 1 and 2. For specificity testing, DNA concentrations in all samples were normalized to 10 ng/µL. The quantitative PCR experiment was conducted in triplicate using the protocol described previously in Section 2.6. Furthermore, sensitivity tests on serially diluted DNA concentrations were performed. The DNA-based sensitivity was tested by 6 tenfold serial dilutions starting from DNA extracted from 10 × 10^−5^ to 10 × 10^−11^. All tests were carried out in triplicate using the quantitative PCR protocol described above. The reaction efficiency was estimated by building reference standard curves obtained between the Ct values and logarithmic DNA dilutions. The linear regression was calculated using the QIA-quant software (Qiagen, Hilden, Germany). The slope and R squared values were computed using the same software as well.

### 2.7. Statistical Analyses

Data were described using exponential values and the statistical treatment has been performed on the values transformed in Log10. The standard curve was built using the QIA-quant 96 software (Qiagen, Hilden, Germany). Statistics were performed by NCSS 2020 v 20.0.2 software (NCSS Statistical Software, East Kaysville, UT, USA) for the Bland–Altman plot to test the agreement between methods and Minitab 19 software (GMSL, Milan, Italy) for the One-way ANOVA analysis.

## 3. Results

### 3.1. Plate Count Enumeration of Food Supplements

*Lactobacilli* and *Bifidobacteria* enumeration of food supplements 1 and 2, were expressed as CFU/g values and are given in Table 3 and Table 4. Considering that bacteria dilutions from 10 × 10^−2^ to 10 × 10^−7^ were too numerous to be directly counted by the operator, only colonies grew up from 10 × 10^−8^, 10 × 10^−9^, and 10 × 10^−10^ were evaluated for each sample. Probiotic enumeration of food supplements 1 (*Lactobacillus* spp. and *Bifidobacterium* spp.) and 2 (every single component, *B. animalis* spp. *lactis*, *B. breve*, *L. rhamnosus,* and *L. paracasei*) were compliant with batch specifications defined by the supplier.

### 3.2. Flow-Cytometric Analysis: Verification of Physiological States

**Figure 2:**
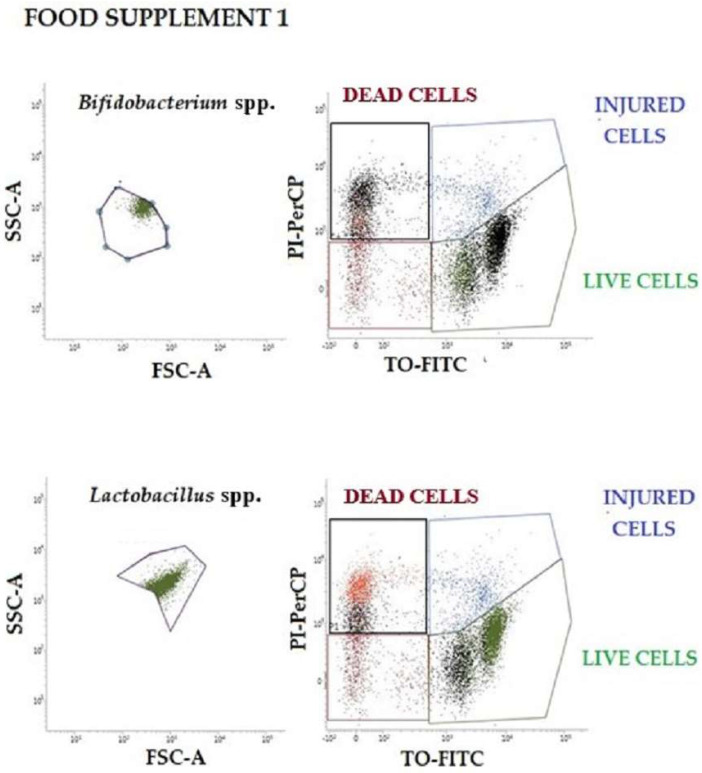
Representative dot-plots of food supplement 1 were analyzed by flow cytometry. Two different graphs are shown for both *Bifidobacterium* spp. and *Lactobacillus* spp.: one represents the morphological features of each population, while the other on the right represents bacteria vitality, (red dots are dead cells; blue dots are injured cells; green dots are live cells). SSC-A: Side Scatter-Area; FSC-A: Forward Scatter-Area; PI-PerCP: Propidium Iodide-Peridinin-Chlorophyll-Protein; TO-FITC: Thiazole Orange-Fluorescein isothiocyanate.

**Figure 3:**
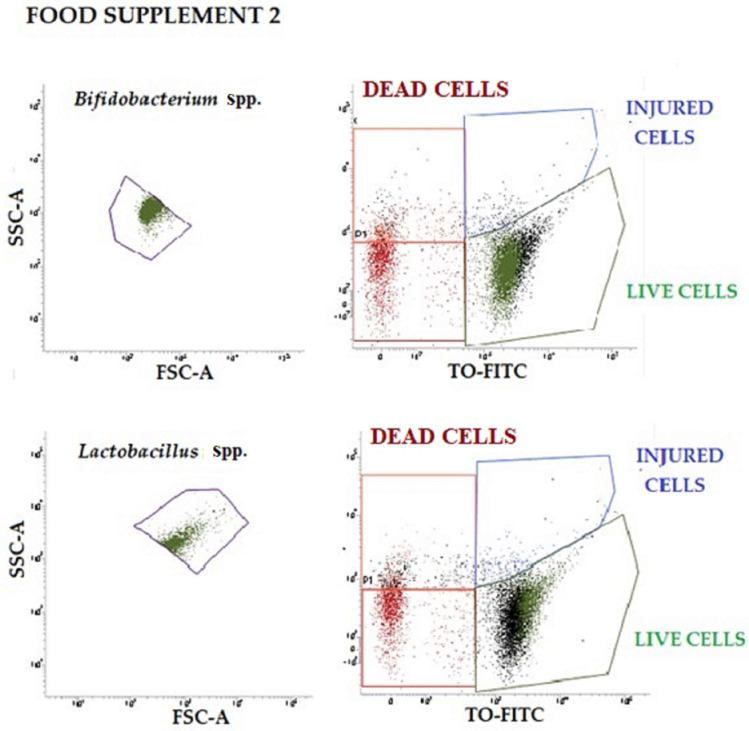
Representative dot-plots of food supplement 2 analyzed by flow cytometry. Two different graphs are shown for both *Bifidobacterium* spp. and *Lactobacillus* spp.: one represents the morphological features of each population, while the other on the right represents bacteria vitality, (red dots are dead cells; blue dots are injured cells; green dots are live cells). SSC-A: Side Scatter-Area; FSC-A: Forward Scatter-Area; PI-PerCP: Propidium Iodide-Peridinin-Chlorophyll-Protein; TO-FITC: Thiazole Orange-Fluorescein isothiocyanate.

To verify the physiological states of probiotics, the results of plate count were compared with flow-cytometry data, following double staining with TO (viable cells) and PI (dead cells). The vitality of subpopulations found in food supplements 1 and 2 was identified based on the different localization taken in the four quadrants of the TO-PI dot-plot graphs (shown in Figure 2 and Figure 3). The injured population can often be observed intermediated between the live and dead populations (blue gate). Only live cell populations can proliferate and generate colonies on plates (green plot). The number of proliferating cells has been used for the statistical evaluation and for the comparison with plate count and RT-qPCR in food supplement 2. The dual-parameter dot plots shown in Figure 2 and Figure 3 indicate the existence of two main subpopulations of probiotics that exhibit dynamic changes in culture (live and damaged). Although heterogeneity in pure culture is common, population complexity and vitality are rarely reported affecting the analytical results.

In Table 5 below, an enumeration of *Lactobacillus* spp., *Bifidobacterium* spp., and the total count of bacteria obtained from flow cytometry of food supplement 1 are reported.

In Table 6 below, *Lactobacillus* spp. counts, *Bifidobacterium* spp. counts, and total count of bacteria obtained from flow cytometry on food supplement 2 are reported.

### 3.3. Optimization of the Strain-Specific qPCR System on Food Supplement 1

The optimization of the RT-qPCR condition was performed using food supplement 1, which is composed of seven different probiotic strains. The presence of multiple species allowed us to define the best reactions to qualitatively discriminate between strains, especially in terms of annealing temperature. To confirm whether primer pairs were species-specific for the identification of each *Lactobacillus* and *Bifidobacterium* species, RT-qPCR assays were tested on all strains contained in the food supplement mixture. For each of the primer pairs, the amplification product was exclusive with high specificity. The results confirmed 100% specificity for all species. All DNA from *Lactobacillus* and *Bifidobacterium* species yielded detectable amplicon, whereas none of the non-target species generated any signals at all. The Ct ranges were 13 to 36 for each species. Thus, all primer pairs were considered specific and sensitive for the detection of any individual species of probiotic in the mixture.

These preliminary tests allowed a full verification of all the species declared by the supplier and the certification of their presence for a quality control intent. This implementation with RT-qPCR greatly improves the performance of the current methods employed in the laboratory. Plate count is limited by the identification of colony morphology (only the two main populations are discriminated), while flow cytometry, although having the advantage of distinguishing bacterial viability, does not permit a single species-strains differentiation. The 100% sensitivity of the RT-qPCR method and its low LOD offer the possibility to analyze samples without the need for an enrichment step in culture, saving time in the release of the product.

### 3.4. Validation of the Strain-Specific qPCR System: Standard Curve Creation

**Figure 4:**
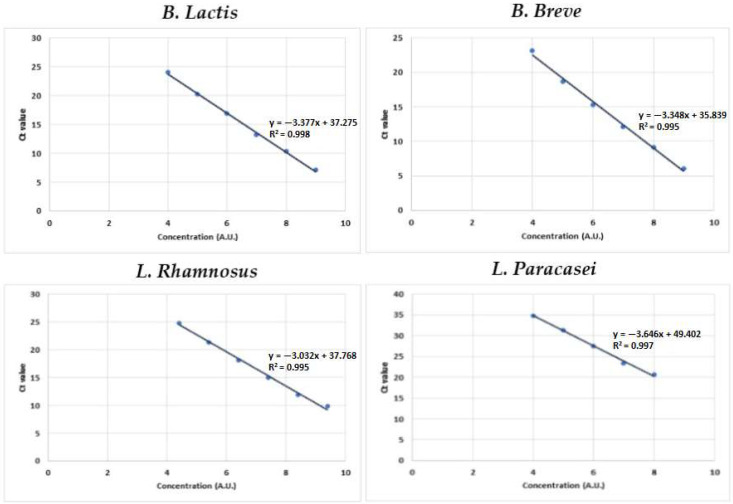
Constructed standard curves imported into different qPCR runs and used for the quantification of DNA copies from unknown extracted samples of food supplement 2. The curves were obtained by plotting the mean values of the Log-calculated concentration of known samples versus the cycle threshold value (Ct). A.U.: Arbitrary Units; qPCR: quantitative polymerase chain reaction.

To correlate quantitative data with colony-forming units, DNA was extracted from 1 mL of *L. rhamnosus*, *L. paracasei*, *B. animalis* spp. *lactis* and *B. breve* individually and examined using RT-qPCR. The linear regression, representative of six different concentrations for each strain, was built for each specific pair of primers tested on food supplement 2 using the QIA-quant 96 software. The standard curves derived from the cycle threshold values (Ct) are shown in Figure 4. Concentrations were expressed in arbitrary units (AI). Logarithms (base 10) of concentrations were plotted against crossing points. The optimal threshold was chosen automatically by the software. The script examined different threshold positions, calculating the coefficient of determination (r2) for each resulting standard curve. The maximum coefficient of determination pointed to the optimal threshold (typically, the maximum r2 was larger than 99%). The optimal threshold was used to calculate Ct for the unknown sample obtained by the extractions of food supplement 2. All primer pairs exhibited a linear relationship over the range 10 × 10^−2^ to 10 × 10^−7^. The slopes for the specific primer pairs of *B. lactis*, *B. breve*, *L. rhamnosus,* and *L. paracasei* were −3.377, −3.348, −3.032, and −3.636, respectively, and the *R*^2^ values were 0.998, 0.995, 0.995, and 0.997, respectively (Figure 4).

### 3.5. Derivation of Values from Crossing Points for qPCR Quantification of Food Supplement 2 Species

The DNA extracted from three sachets of food supplement 2 (all in duplicate) was profiled by RT- qPCR using the specific primers described in the previous paragraphs. To obtain absolute quantification of the extracts, Ct values given from the amplification were interpolated with those of the standard curves created. Briefly, RT-qPCR results demonstrated higher quantities of probiotic strains than those declared by the supplier, probably due to the method’s high sensitivity and specificity in identified single species. Data regarding RT-qPCR enumeration of *L. rhamnosus*, *L. paracasei*, *B. animalis* spp. *lactis,* and *B. breve* are reported in Table 7 and Table 8 below.

Another hypothesis to explain the over-estimation of probiotics by RT-qPCR could be the imprecise initial count of raw material calculated by the plate method or the fact that qPCR identifies the molecular signature of strains independently from the physiological state.

### 3.6. Statistical Validation of Results: Comparison between Plate Count, Flow Cytometry, and RT-qPCR in Food Supplement 2

**Figure 5:**
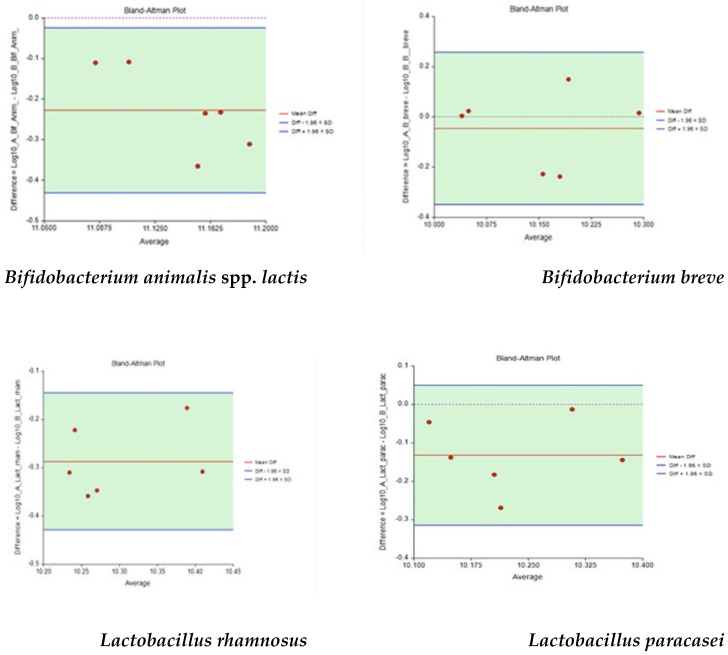
Bland–Altman plots for data from Table 7, with the representation of the limits of agreement (blue line) from −1.96 × SD to 1.96 × SD. SD: Standard Deviation; Diff: difference.

The Bland–Altman plot (mean difference or agreement limits) has been used to compare two measurements of the same probiotic strain of food supplement 2 by RT-qPCR. For all probiotics (B. *animalis* spp. *lactis*, *B. breve*, *L. rhamnosus,* and *L. paracasei*) no outliers were found during the comparison of measurements, as reported in Figure 5. Data presented in each plot were distributed with random variability and demonstrated that the two methods could be equally applicable to all probiotics. Bias, in terms of mean difference and the limits of agreement, at the 95% confidence intervals for each quantity, were evaluated for every calculation.

The comparison between the three methods tested in this work in enumerating *Lactobacillus* spp. vs. *Bifidobacterium* spp. is reported in Table 9 and Table 10.

**Figure 6:**
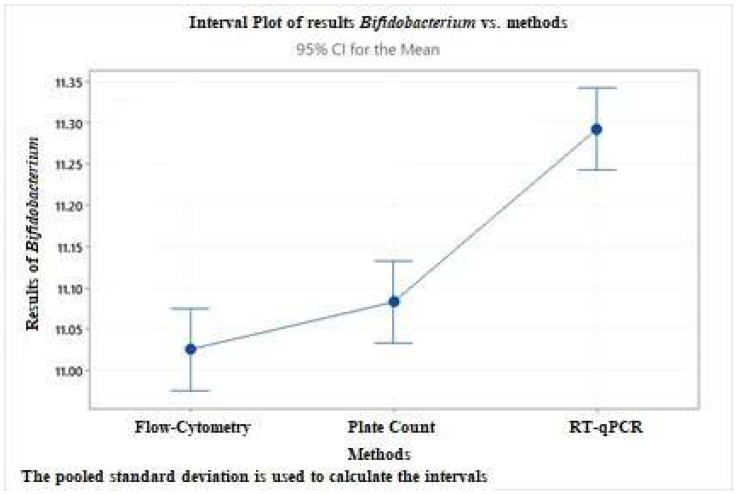
Interval graph representing the difference in counting between *Bifidobacterium* spp. count in food supplement 2 obtained from the three methods tested. CI: confidence interval.

**Figure 7:**
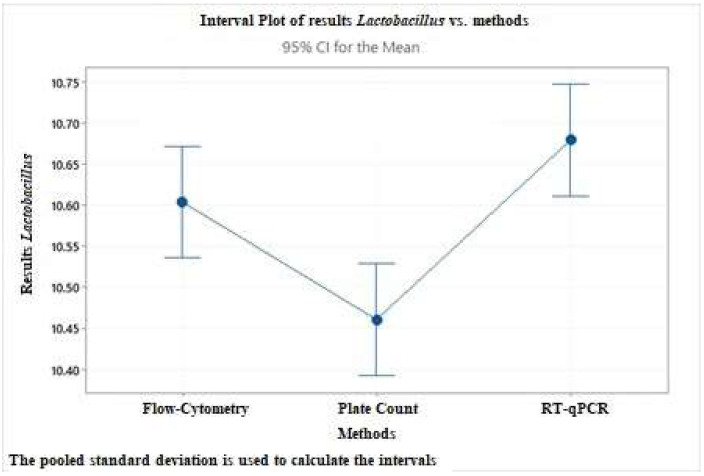
Interval graph representing the difference in counting between *Lactobacillus* spp. count in food supplement 2 obtained from the three methods tested. CI: confidence interval.

A one-way ANOVA test has been performed to establish the three methods’ agreement for *Bifidobacterium* spp. and *Lactobacillus* spp. counts. The following graphs (Figure 6 and Figure 7) are representative of the differences between *Bifidobacterium* spp. *and Lactobacillus* spp. estimation in food supplement 2 by the three methods.

The quantification of probiotic strains obtained by the three methods are significantly different (*p* ≤ 0.05) from each other, and RT-qPCR seems to give results higher than the other two methods. Using RT-qPCR, single-copy genetic targets, each representing a single cell and not colonies, provide more precise absolute enumeration. In addition, the amplification of genetic targets in damaged cells or present extracellularly could influence an in-excess target quantity count.

## 4. Discussion

The establishment of new quality control methods to enumerate raw materials in food supplements can help in overcoming some of the current limitations of the microbiological-based probiotic industry [9,13,24]. The described method based on RT-qPCR and flow cytometry could contribute significantly to improving the current methods available [16,18,38,39,40]. In routine laboratories, culture methods are considered the gold standard for quality control assurance, but it is a time-consuming, operator-dependent, and cost-effective test. RT-qPCR is considerably faster and has lower variability. Another key advantage of qPCR is the detection of a strain-specific DNA target, which can be easily developed for any species based on the unique genetic identity [14,22,37]. Current culture methods rely on growth using general and selective media, which only detect probiotics that can create a colony [9,42,43,44].

Concerning costs, the implementation of new technologies, such as flow cytometry and RT-qPCR, is a long-term, cost-effective solution in the management of a high number of samples per day compared to probiotic cultures [23,30,34]. These methods provide the optimal balance of costs between reagents and man-hours, representing a suitable solution in routine testing.

The importance of identifying specific probiotic strains is the peculiar ability of each to produce health benefits in a human environment [1,5,44]. This is an important issue with classical microbiology methods. The inability to identify specific strains from one another can be overcome by genetic tests [14,22]. RT-qPCR can be used to distinguish and quantify very similar strains of probiotics using their variability in gene sequences, and this technology can be used in combination with flow cytometry to also define probiotics’ vitality [18,23,30,38].

As demonstrated in our work, RT-qPCR can be used as a qualitative test (e.g., in food supplement 1) and as a quantitative test (food supplement 2). The results obtained from the quantification of food supplement 2 have demonstrated that RT-qPCR can be used to absolute the enumerate probiotic cells at the strain level for the dietary supplement industry. This technology is more sensitive for strain-specific detection with less operator variability than the current plate count enumeration. In our case, the quantification by RT-qPCR overestimates the number of probiotics declared by the producer and count by culture method, probably due to its higher sensitivity in also identifying single cells and for the amplification of genetic targets as well as dead and damaged cells.

New experiments may need to be implemented in order to create a unique molecular assay able to simultaneously detect strains and cell viability using specific DNA markers. To now, a combined approach based on flow cytometry and RT-qPCR could represent an optimal way to perform quality control assessments for product release and overcome classical microbiological methods.

## 5. Conclusions

The assay developed for strain-specific detection of probiotics in food supplements showed a high degree of specificity, sensitivity, efficiency, and low variance between measurements. The approach can be performed in combination with flow cytometry and used in the quality control assessment of multi-strain dietary products. This approach, based on RT-qPCR, provides a feasible method for strain-specific identification and enumeration in food supplements for managing quality control release and quality assurance overcoming some of the limitations of the common microbiological methods. In conclusion, we develop strain-specific RT-qPCR to quantify *L. rhamnosus*, *L. paracasei*, *B. animalis* spp. *lactis,* and *B. breve* in food supplements without the needing for culture enrichment steps. RT-qPCR is a sensitive and rapid tool to determine low abundant bacterial strains in samples with mixed microbial populations and to implement with flow-cytometry to obtain a full characterization of multi-strain food supplements.

## Figures and Tables

**Table 1:** Primer sequences used in the RT-qPCR assay for the detection of probiotic strains in food supplement 1.

Probiotic Strain-Primer Name		Sequence
*Bifidobacterium longum*	FW	5′-TTC CAG TTG ATC GCA TGG TC-3′
*Bifidobacterium longum*	RV	5′-TTC CAG TTG ATC GA TGG TC-3′
*Bifidobacterium longum* spp. Infantis *	FW	5′-TTC CAG TTG ATC GCA TAA TC-3′
*Bifidobacterium longum* spp. Infantis *	RV	5′-GAA ACC CCA TCT CTG GGA TC-3′
*Lactobacillus paracasei*	FW	5′-ACA TCA GTG TAT TGC TTG TCA GTG AAT AC-3′
*Lactobacillus paracasei*	RV	5′-CC TGC GGG TAC TGA GAT GTT TC-3′
*Lactobacillus acidophilus*	FW	5′-GAA AGA GCC CAA ACC AAG TGA TT-3′
*Lactobacillus acidophilus*	RV	5′-CTT CCC AGA TAA TTC AAC TAT CGC TTA-3′
*Lactobacillus plantarum*	FW	5′-TGG ATC ACC TCC TTT CTA AGG AAT-3′
*Lactobacillus plantarum*	RV	5′-TGT TCT CGG TTT CAT TAT GAA AAA ATA-3′
*Bifidobacterium breve*	FW	5′-CCG GAT CGT CCA TCA CAC-3′
*Bifidobacterium breve*	RV	5′-ACA AAG TGC CTT GCT CCC T-3′
*Lactobacillus delbrueckii* **	FW	5′-CAC TTG TAC GTT GAA AAC TGA ATA TCT TAA-3′
*Lactobacillus delbrueckii* **	RV	5′-CGA ACT CTC TCG GTC GCT TT-3′

* Reclassified as *Bifidobacterium animalis* spp. Lactis. ** Reclassified as *Lactobacillus helveticus*. FW: forward; RV: reverse; RT-qPCR: real-time quantitative polymerase chain reaction.

**Table 2:** Primer sequences used in the RT-qPCR assay for the detection and quantification of probiotic strains in food supplement 2.

Probiotic Strain		Sequence
*Bifidobacterium animalis* spp. *lactis*	FW	5′-ACCTCACCAATCCGCTGTTC-3′
*Bifidobacterium animalis* spp. *lactis*	RV	5′-GATCCGCATGGTGGAACTCT-3′
*Bifidobacterium breve*	FW	5′-CCG GAT CGT CCA TCA CAC-3′
*Bifidobacterium breve*	RV	5′-ACA AAG TGC CTT GCT CCC T-3′
*Lactobacillus rhamnosus*	FW	5′-GGC GTC CTG CTA GTC CTA AT-3′
*Lactobacillus rhamnosus*	RV	5′-GCA GGA TAA GCC GAT ACT TC-3′
*Lactobacillus paracasei*	FW	5′-CAA CCG TGA TGA CAC TG-3′
*Lactobacillus paracasei*	RV	5′-CCA ACG TTA ATC CGG TAC TG-3′

FW: forward; RV: reverse.

**Table 3:** Results obtained from plate count method of food supplement 1.

Sample	*Lactobacillus* spp. (CFU/g)	*Bifidobacterium* spp. (CFU/g)
Premix	2.32 × 10^11^	2.12 × 10^11^
Sachet—batch A	6.15 × 10^10^	6.10 × 10^10^
Sachet—batch B	3.95 × 10^10^	6.50 × 10^10^
Sachet—batch C	6.30 × 10^10^	6.20 × 10^10^

CFU: Colony-forming Unit.

**Table 4:** Results obtained from plate count method of food supplement 2.

Sample	*Bifidobacterium animalis* spp. *lactis* (CFU/g)	*Bifidobacterium breve* (CFU/g)	*Lactobacillus rhamnosus* (CFU/g)	*Lactobacillus paracasei* (CFU/g)
Sachets	1.13 × 10^11^	1.10 × 10^10^	1.20 × 10^10^	1.25 × 10^10^

**Table 5:** Probiotic counts of food supplement 1 obtained by flow cytometry.

Sample	*Lactobacillus* spp. (FU/g)	*Bifidobacterium* spp. (FU/g)	Total Count (FU/g)
Premix	2.12 × 10^11^	2.32 × 10^11^	4.44 × 10^11^
Sachet—batch A	6.15 × 10^10^	6.14 × 10^10^	1.23 × 10^11^
Sachet—batch B	4.61 × 10^10^	6.29 × 10^10^	1.09 × 10^11^
Sachet—batch C	6.28 × 10^10^	6.22 × 10^10^	1.25 × 10^11^

FU: Fluorescence Unit.

**Table 6:** Probiotic counts of food supplement 2 obtained by flow cytometry.

Sample	*Lactobacillus* spp. (FU/g)	*Bifidobacterium* spp. (FU/g)	Total Count (FU/g)
Sachet 1	4.26 × 10^10^	1.08 × 10^11^	1.51 × 10^11^
Sachet 2	4.03 × 10^10^	1.08 × 10^11^	1.48 × 10^11^
Sachet 3	2.14 × 10^10^	1.02 × 10^11^	1.23 × 10^11^
Sachet 4	4.06 × 10^10^	1.05 × 10^11^	1.46 × 10^11^

**Table 7:** Count results by RT-qPCR of food supplement 2 expressed as Log10 for *Bifidobacterium animalis* spp. *lactis,* and *Bifidobacterium breve*.

Sample	Probiotic Strains							
	*B. animalis* spp. *Lactis*				*B. breve*			
	Plate Count (A) CFU/g	Log_10_ (A)	RT-qPCR (B) CFU/g	Log_10_ (B)	Plate Count (A) CFU/g	Log_10_ (A)	RT-qPCR (B) CFU/g	Log_10_ (B)
Stick 1	1.13 × 10^11^	11.0531	1.45 × 10^11^	11.1614	1.10 × 10^10^	10.414	1.09 × 10^10^	10.0374
Stick 1	1.07 × 10^11^	11.0294	1.38 × 10^11^	11.1399	1.15 × 10^10^	10.0607	1.09 × 10^10^	10.0374
Stick 2	9.36 × 10^10^	10.9713	2.17 × 10^11^	11.3365	1.10 × 10^10^	10.0414	1.86 × 10^10^	10.2695
Stick 2	1.08 × 10^11^	11.0334	2.21 × 10^11^	11.3444	1.15 × 10^10^	10.0607	1.99 × 10^10^	10.2989
Stick 3	1.10 × 10^11^	11.0414	1.89 × 10^11^	11.2765	2.00 × 10^10^	10.3010	1.93 × 10^10^	10.2856
Stick 3	1.13 × 10^11^	11.0531	1.93 × 10^11^	11.2856	1.85 × 10^10^	10.2672	1.31 × 10^10^	10.1173

**Table 8:** Count results by RT-qPCR of food supplement 2 expressed as Log10 for *Lactobacillus rhamnosus* and *Lactobacillus paracasei*.

Sample	Probiotic Strains							
	*L. rhamnosus*				*L. paracasei*			
	Plate Count (A) CFU/g	Log_10_ (A)	RT-qPCR (B) CFU/g	Log_10_ (B)	Plate Count (A) CFU/g	Log_10_ (A)	RT-qPCR (B) CFU/g	Log_10_ (B)
Stick 1	1.20 × 10^10^	10.0792	2.45 × 10^10^	10.3892	1.25 × 10^10^	10.0969	1.39 × 10^10^	10.1430
Stick 1	1.35 × 10^10^	10.1303	2.25 × 10^10^	10.3522	1.30 × 10^10^	10.1139	1.98 × 10^10^	10.2967
Stick 2	1.25 × 10^10^	10.0969	2.78 × 10^10^	10.4440	1.20 × 10^10^	10.0792	2.23 × 10^10^	10.3483
Stick 2	2.00 × 10^10^	10.3010	3.00 × 10^10^	10.4771	2.00 × 10^10^	10.3010	2.79 × 10^10^	10.4456
Stick 3	1.80 × 10^10^	10.2553	3.66 × 10^10^	10.5635	2.00 × 10^10^	10.3010	2.06 × 10^10^	10.3139
Stick 3	1.20 × 10^10^	10.0792	2.47 × 10^10^	10.4378	1.20 × 10^10^	10.792	1.65 × 10^10^	10.2175

**Table 9:** Measurements expressed as Log10 for *Bifidobacterium* spp. in food supplement 2, obtained by the three methods tested.

Sample	Probiotics Strains					
	*Bifidobacterium* spp.					
	Plate Count (A) CFU/g	Log_10_ (A)	RT-qPCR (B) CFU/g	Log_10_ (B)	Flow Cytometry (C) FU/g	Log_10_ (C)
Stick 1	1.24 × 10^11^	11.0934	1.56 × 10^11^	11.1928	1.08 × 10^11^	11.0334
Stick 1	1.19 × 10^11^	11.0737	1.49 × 10^11^	11.1729	9.61 × 10^10^	10.9827
Stick 2	1.05 × 10^11^	11.0195	2.36 × 10^11^	11.3722	1.08 × 10^11^	11.0334
Stick 2	1.20 × 10^11^	11.0774	2.41 × 10^11^	11.3818	1.09 × 10^11^	11.0374
Stick 3	1.30 × 10^11^	11.1139	2.08 × 10^11^	11.3187	1.05 × 10^11^	11.0212
Stick 3	1.32 × 10^11^	11.1189	2.06 × 10^11^	11.3141	1.11 × 10^11^	11.0453

**Table 10:** Measurements expressed as Log10 for *Lactobacillus* spp. (Table 10) in food supplement 2, obtained by the three methods tested.

Sample	Probiotics Strains					
	*Lactobacillus* spp.					
	Plate Count (A) CFU/g	Log_10_ (A)	RT-qPCR (B) CFU/g	Log_10_ (B)	Flow Cytometry (C) FU/g	Log_10_ (C)
Stick 1	2.45 × 10^10^	10.3892	3.84 × 10^10^	10.5843	4.26 × 10^10^	10.6294
Stick 1	2.65 × 10^10^	10.4232	4.23 × 10^10^	10.6263	3.22 × 10^10^	10.5079
Stick 2	2.45 × 10^10^	10.3892	5.01 × 10^10^	10.6998	4.03 × 10^10^	10.6053
Stick 2	4.00 × 10^10^	10.6021	5.79 × 10^10^	10.7627	4.24 × 10^10^	10.6274
Stick 3	3.80 × 10^10^	10.5798	5.72 × 10^10^	10.7574	4.06 × 10^10^	10.6085
Stick 3	2.40 × 10^10^	10.3802	4.39 × 10^10^	10.6425	4.39 × 10^10^	10.6425

## Data Availability

The authors confirm that all relevant data are included in the article and materials are available on request.
